# Medical Register and Directory of the United States

**Published:** 1873-04

**Authors:** S. W. Butler

**Affiliations:** 115 S. Seventh Street, Philadelphia, Pennsylvania


*Medical Register and Directory of the United States.*

Work on this important publication, which was delayed by illness, is now resumed with energy, and it will be issued as soon as the vast amount of material can be collected and arranged. The *Register and Directory* will contain as complete a list of the medical men in the United States, and their professional status, as can be obtained by personal application to each, and from other sources. Also, a complete medical history of each State, including all its medical institutions, societies, etc., and all medical legislation. Nothing will be omitted that will be of possible benefit to the profession. The coöperation of medical men in every section of the country is earnestly solicited in replying to the circulars sent out, and in *giving brief outlines of the history of medical institutions, hospitals, colleges, etc., and of State, and the more important local medical organizations.*

Circulars will be sent out hereafter *by States*, and announced through the medical journals. They *have been sent* to the following States and Territories, viz.: Alabama, Arizona, Arkansas, California, Colorado, Connecticut, Dakotah, Delaware, District of Columbia, Florida and Georgia. In *March*, they will be sent to Illinois, Indiana, Iowa, Kansas, Kentucky and Louisiana, and all the remaining Territories. IMMEDIATE ANSWERS are requested, and prompt notification if circulars are not received.

Physicians are particularly requested to make as full replies as possible to the questions in the circular.

S. W. BUTLER, M.D.,
115 S. Seventh Street, Philadelphia, Pennsylvania.

